# Synthesis and characterization of some new Schiff base azo disperse dyes based on chromene moiety for simultaneous dyeing and antimicrobial finishing

**DOI:** 10.1038/s41598-024-73253-7

**Published:** 2024-10-05

**Authors:** Hagar Fathy, M. H. Helal, Dina Abbas, Fatma A. Mohamed

**Affiliations:** 1 Dyeing, Printing and Auxiliaries Department, Institute for Textile Research and Technology, National Research Center, 33 El Buhouth St., Dokki, Giza, 12622 Egypt; 2 Chemistry Department, Faculty of Science, Helwan University, Cairo, Egypt

**Keywords:** Nylon, Polyester, Fabric, Azo disperse dyes, Schiff base, Dyeing, Biochemistry, Chemical biology, Medical research

## Abstract

New azo Schiff base disperse dyes based on a chromene moiety were synthesized by reacting (2-amino-7-hydroxy-4-(4-methoxyphenyl)-4 H-chromene-3 carbonitrile) and(2-amino-4-(3,4-dimethoxyphenyl)-7-hydroxy-4 H-chromene-3-carbonitrile), with vanillin and ninhydrin, producing new chromene Schiff base derivatives, which in turn were coupled with 2-chloro-4-nitroaniline diazonium salt to give new 4 azo disperse dyes (1–4). The structures of the prepared dyes were confirmed using elemental analysis, ^1^HNMR spectroscopy, mass spectrometry, and IR. The synthesized dyes were applied to polyester and nylon fabrics using different dyeing techniques: high temperature- high pressure, and ultrasonic dyeing methods. The highest K/S values for all investigated dyes were achieved usinga high temperature-high pressure dyeing technique. Also, the color reflectance of all synthesized dyes with different dyeing shades (1%, 2%, and 3%) was obtained. The fastness properties of the dyed samples using the investigated dyes showed good color fastness toward light, washing, rubbing, and perspiration fastness. The presence of a chromene moiety and Schiff base in the investigated dyes promotes a higher antimicrobial activity on nylon and polyester fabrics against all tested bacteria (E. coli gram-negative and Staphylococcus aureus gram-positive) and two fungi, Aspergillus Niger and Candida albicans.

## Introduction

In recent years, azo-functionalized dyes bearing aromatic heterocyclic components have attracted attention as they achieve simultaneous dyeing with numerous biological characteristics and are used in many different sectors, including medical applications^1,2^, food, textile industries, cosmetics, and so on^3^.

Heterocyclic moiety has gained attention in dye chemistry, especially disperse dye, due to its superior build-up profile, strong tinctorial, brighter hues, and superior fastness qualities compared to simple carbocyclic systems^4^. Besides displaying an important range of biological activity, including antimicrobial, anti-inflammatory, anti-malarial, anti-oxidant, anti-tumor, and anti-cancer properties, in addition to their critical role in many industrial applications^5^.

Methoxyphenyl-4 H-chromene-3-carbonitrile derivatives represent an important class of heterocyclic azo dyes that are well-known for their extensive use as functional colorants^6,7^. The chromene moiety is one of the most common heterocyclic systems, comprising a phenyl ring fused with an oxine ring^8^. The incorporation of chromene with an azo linkage enhances their dyeing and biological performance^9^ and that of other useful chemotherapeutic agents. The chromene moiety gives the dye structure an additional action, such as anti-tuberculosis^10^, anti-diabetic^11^, anti-cholinesterase^12^, antiepileptic^13^, and anti-HIV activity, in addition to the similar characteristics of heterocyclic compounds^14^.

Schiff bases are a great phenomenon that provides advantages in synthesizing heterocyclic dyes with numerous biological characteristics, including antibacterial, antiviral, anticancer, and antioxidant effects^15^. Schiff bases” refer to a class of organic compounds resulting from the condensation reaction between a primary amine and a carbonyl compound, typically an aldehyde or a ketone^16^. Many heterocyclic Schiff base compounds exhibit more promising physical, and chemical properties than their heterocyclic analogs^17,18^.In the past few years, many reviews have been published, focusing on the biological activity of heterocyclic Schiff base moiety^19,20^.

For all the previous importance of heterocyclic chromene dyes, herein, we present the synthesis, characterization, and antimicrobial activity of some new Schiff-base azo disperse dyes based on chromene moiety and their application on synthetic fabrics(nylon and polyester).

## Experimental

### Materials

#### Fabrics

100% polyester fabric that has been bleached and scoured (160 g/m2; Misr El- Mahalla Co., Egypt); El Mahalla El-Kubra Company provided 100% nylon fabric that has been bleached and scoured (149 g/m2). The fabric was treated with a solution containing nonionic detergent (Hostapal CV, Clariant-Egypt, 5 g/L) and sodium carbonate (2 g/L) in a 50:1 ratio at 60 °C for 30 min before application. It was then completely rinsed with water and allowed to air dry at room temperature.

#### Chemicals

HostapalCV (Clariant, Egypt) was used as a nonionic detergent. 4-methoxybenzaldehyde, 3, 4-dimethoxybenzaldehyde, malononitrile, resorcinol, Vanillin, Ninhydrin, and 2-chloro-4-nitroaniline were provided by Sigma Aldrich. All other compounds utilized in this investigation were laboratory reagent grade, without additional purification.

#### Instruments

Melting points were found using the “Gallenkamp” device. Using a “Nicolet 5000 FT-IR” spectrophotometer, IR spectra were recorded. “Bruker WP 400 MHz” was used to produce ^1^H NMR spectra in DMSO-d6. “Quadrupole GC/MS Thermo Scientific Focus/DSQII” was used to calculate the mass spectra at 70 eV.Warmth.

### Synthesis of dyes

#### Synthesis of chromene moiety

**Scheme 1:**
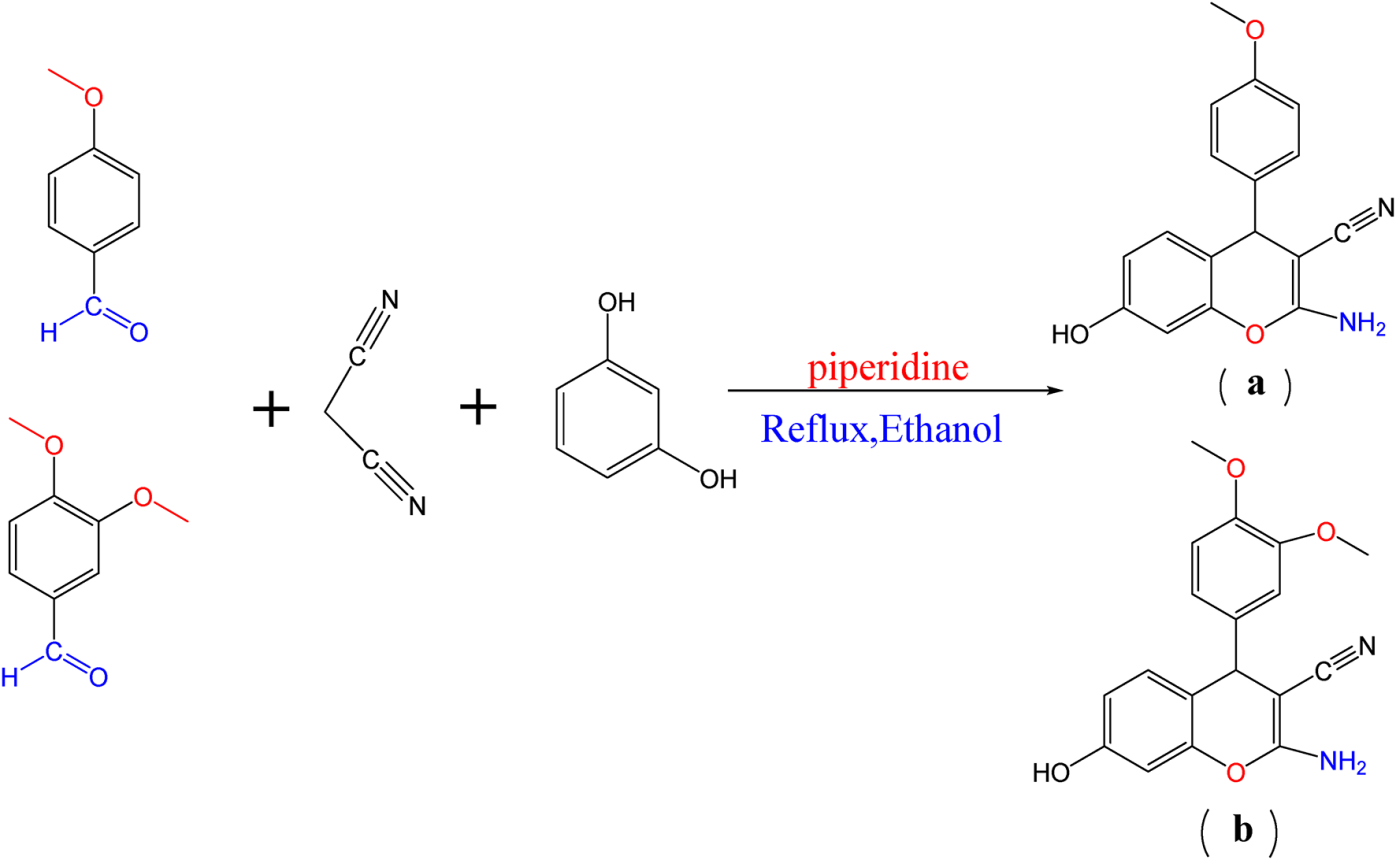
Synthesis of chromene moiety.

A mixture of 4-methoxybenzaldehyde (6.8 ml, 0.05 mol) or 3, 4-dimethoxybenzaldehyde (8.3 g, 0.05 mol), malononitrile (3.3 ml, 0.05 mol), resorcinol (5.5 g, 0.05 mol), ethanol (30 ml), and drops of piperidine was stirred and refluxed until the reaction was completed 2 h (Scheme 1) respectively. The product precipitated when the reaction cooled at room temperature. The crude product was filtered, dried, and recrystallized from ethanol to give (a) and (b) respectively^21^.

##### 2-amino-7-hydroxy-4-(4-methoxyphenyl)-4 H-chromene-3-carbonitrile (a)

Orange crystals m.p.232 C, yield (95.2%), C_17_H_14_N_2_O_3_MS, m/z 294 M^−1^ [293] percent, calcd: C, 69.38; H, 4.76; N, 9.52%, found: C, 69.08; H, 4.70; N, 9.05%. FT-IR (cm^−1^) 3411 (OH), 3335(-NH_2_-), 3220 (C-H, aromatic), 2188 (CN), 1587 (C = C, aromatic), 1400 (C-O of chromene);^1^HNMR (d, ppm), 3.6901 (s, 3 H, CH_3_), 4.5338 (s, H,CH chromene), 6.377–7.057 (m, 7 H, C_6_H_4_, C_6_H_3_), 7.50 (s, 1H, NH_2_), 9.743(s, 1H, OH).

##### 2-amino-4-(3, 4-dimethoxyphenyl)-7-hydroxy-4 H-chromene-3-carbonitrile(b)

Yellow crystals m.p.195 °C, yield (90.2%), C_18_H_16_N_2_O_4_MS, m/z 324 M^+2^[326] percent, calcd: C, 66.67; H,4.94; N, 8.64%, found: C, 66.08; H, 4.77; N, 9.14%. FT- IR (cm^−1^) 3411 (OH), 3335(-NH_2_-), 3220 (C-H, aromatic), 2188 (CN), 1587 (C = C, aromatic), 1400 (C-O of chromene); ^1^HNMR (d, ppm), 3.6901 (s, 6 H, 2CH_3_), 4.5338 (s, H,CH chromene), 6.377–7.057 (m, 7 H, C_6_H_4_, C_6_H_3_), 7.50 (s, 1H, NH_2_), 9.743(s, 1H, OH).

#### Synthesis of Schiff base

**Scheme 2:**
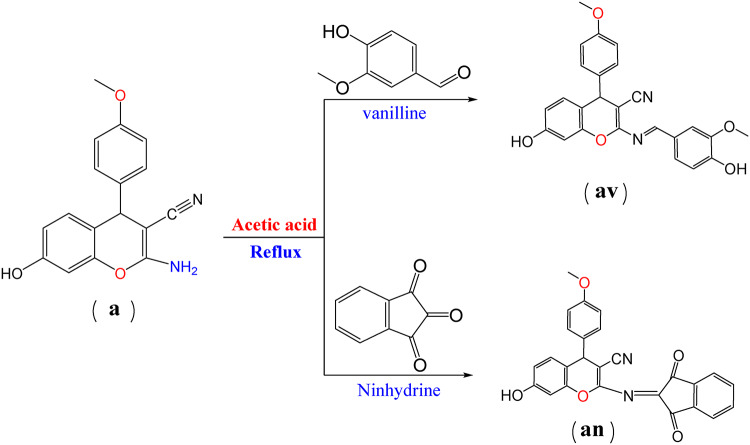
Synthesis of Schiff base with chromene moiety.

**Scheme 3:**
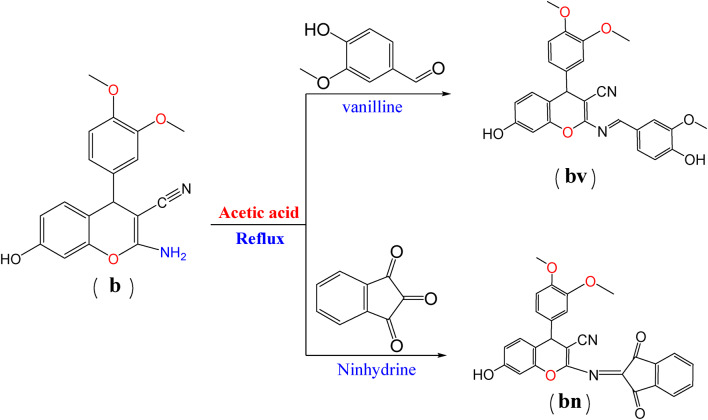
Synthesis of Schiff base with chromene moiety.

A mixture of a (5.88 g, 0.02 mol) or a mixture of b (6.48 g, 0.02 mol), Vanillin (3.04 g, 0.02 mol), or Ninhydrin (3.6 g, 0.02 mol), ethanol (30 ml), and drops of acetic acid was stirred and refluxed until the reaction was completed 4 h (Scheme 2). The product precipitated when the reaction cooled at room temperature. The crude product was filtered, dried, and recrystallized from ethanol to give orange crystals (av), brown crystals (bv), brown crystals (an), and yellow crystals (bn) respectively (Scheme 2, 3).

##### (E)-7-hydroxy-2-((4-hydroxy-3-methoxybenzylidene) amino)-4-(4 methoxyphenyl)-4 H-chromene-3-carbonitrile (av)

Orange crystals m.p.220 °C, yield (95.2%), C_25_H_20_N_2_O_5_ MS, m/z 428 M^+^ [428] percent, calcd: C, 70.08; H, 4.71; N, 6.54%, found: C, 70.03; H, 4.70; N, 6.50%. FT-IR (cm^−1^)3342 (2OH), 2223 (CN), 1559 (C = N, Schiff base), 1440 (C-O); ^1^HNMR (d, ppm), 3.424, 3.811 (s, 6 H, 2OCH_3_), 4.533 (s, H, CH chromone), 6.377–7.0574 (m, 10 H, C_6_H_4_, 2C_6_H_3_), 7.34 (s, 1H, C = N), 9.743 (s, 2 H, 2OH).

##### (E)-4-(3,4-dimethoxyphenyl)-7-hydroxy-2-((4-hydroxy-3methoxybenzylidene) amino)-4 H-chromene-3-carbonitrile (bv)

Brown crystals m.p. 230 °C, yield (90%), C_26_H_22_N_2_O_5_ MS, m/z 458 M^+^ [458] percent, calcd: C, 68.11; H, 4.84; N, 6.11%, found: C, 68.08; H, 4.70; N, 6.05%. FT- IR (cm^−1^)3342 (2OH), 2223 (CN), 1559 (C = N, Schiff base), 1440 (C-O); ^1^HNMR (d, ppm), 3.424, 3.811 (s, 9 H, 3OCH_3_), 4.533 (s, H, CH chromone), 6.377–7.0574 (m, 10 H, C_6_H_4_, 2C_6_H_3_), 7.34 (s, 1H, C = N), 9.743 (s, 2 H, 2OH).

##### 2-((1,3-dioxo-1 H-inden-2(3 H)-ylidene) amino)-7-hydroxy-4-(4-methoxyphenyl)-4 H-chromene-3-carbonitrile (an)

Brown crystals m.p. 250 °C, yield (95.2%) C26H16N2O5 MS, m/z 436 M^+^ [436] percent, calcd: C, 71.56; H, 3.70; N, 6.42%, percent, found: C, 71.50; H, 3.80; N, 6.44%. FT-IR (cm^−1^) 3341 (OH), 2194 (CN), 1641, 1614 (2 C = O) 1587 (C = N), 1400 (C-O); ^1^HNMR (d, ppm), 3.307 (s, 6 H, 2OCH_3_), 4.1906 (s, H, CH chromone), 6.82–7.955 (m, 9 H, 3C_6_H_3_), 11.31 (s, H, OH).

##### 4-(3,4-dimethoxyphenyl)-2-((1,3-dioxo-1 H-inden-2(3 H)-ylidene) amino)-7-hydroxy-4 H-chromene-3-carbonitrile (bn)

Yellow crystalsm.p. 145 °C, yield(88.2%),C_27_H_18_N_2_O_6_ MS, m/z 466 M^+1^[467] percent, calcd: C, 69.52; H, 3.89; N, 6.01%, percent, found: C, 69.08; H,3.99; N, 6.05 FT-IR (cm^−1^) 3341 (OH), 2194 (CN),1641, 1614 (2 C = O) 1587 (C = N), 1400 (C-O); ^1^HNMR (d, ppm), 3.307 (s, 6 H, 2OCH_3_), 4.1906 (s, H, CH chromone), 6.82–7.955 (m, 9 H, 3C_6_H_3_), 11.31 (s, H, OH).

#### General procedure for the synthesis of azo disperse dyes

**Scheme 4:**
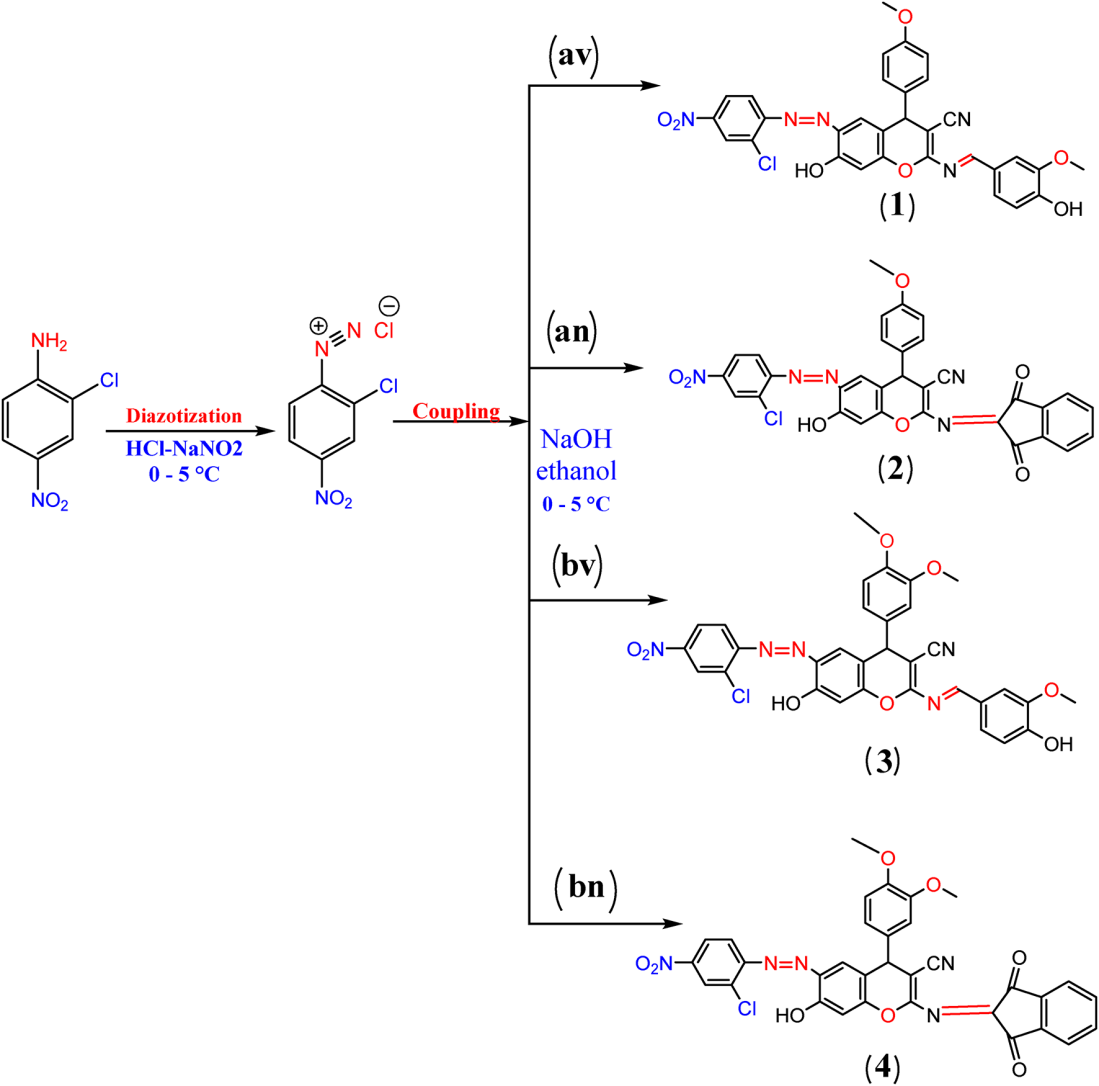
Synthesis azo disperse dyes 1–4.

To an ice-cold solution of the appropriate aromatic amine (2-chloro-4-nitroaniline) (1.72 g, 0.01 mmol) in a concentrated hydrochloric acid (3 mL), a cold aqueous solution of sodium nitrite (1 g), was added dropwise through 15 min at 0–5 °C. The solution was stirred vigorously with traces of sulfamic acid to remove any excess nitrous for 30 min to yield the diazonium salt solution. The diazonium salt solution was then added drop wisely to the pre-cooled solution of compound av, bv, an, bn (0.01 mol, in 20 mL ethanol and 5 mL of 10% NaOH) over 1 h with vigorous stirring and adding sodium acetate to collect the dye particles. Following the reaction’s conclusion, the products were filtered out, cleaned in water to remove any remaining acid, and then dried at room temperature to yield dyes 1–4, which were recrystallized from the appropriate solvent (Scheme 4). The synthesized compounds1-4 with their physical and spectral data are listed below.

Dye**1**: -**6-((2-chloro-4-nitrophenyl) diazenyl)-7-hydroxy-2-((E)-(4-hydroxy-3 methoxybenzylidene)amino)-4-(4-methoxyphenyl)-4 H-chromene-3-carbonitrile**.

Reddish brown powder, Yield = 93.5%, m.p. 100 °C, C_31_H_22_N_5_O_7_Cl MS, m/z 611.5 M^+1^[612] percent, calcd: C, 60.84; H, 3.62; N, 11.44%, found: C, 60.60; H, 3.70; N, 11.50%. FT-IR (cm^−1^)3278 (2OH), 2190 (CN), 1588 (C = N), 1507 (N = N), 1462 (C-O); ^1^HNMR (d, ppm), 3.69 (s, 6 H, 2OCH_3_), 4.53 (s, H, CH chromone), 6.37–7.04 (m, 12 H, C_6_H_4_, 2C_6_H_3_, C_6_H_2_), 7.057 (s, 1H, Sciff base C = N), 9.74 (s, 2 H, 2OH)^13^C NMR (101 MHz, DMSO*d*6) δ (ppm): 29.9, 55.8, 56.1, 77.9, 105.7,112.1, 113.2, 114.8, 114.8, 117.3, 119.8, 122.3, 122.9, 124.5, 125.3, 125.8, 127.3, 129.2, 129.2, 129.4,132.6, 149.3,150.0, 150.4, 151.0, 151.5, 156.7, 158.1, 163.7, 170.8.

Dye **2: -6-((2-chloro-4-nitrophenyl) diazenyl)-2-((1**,**3-dioxo-1 H-inden-2(3 H)-ylidene) amino)-7-hydroxy-4-(4-methoxyphenyl)-4 H-chromene-3-carbonitrile**.

Brown powder, m.p. 115 °C, yield (83%) C_32_H_18_N_5_O_7_Cl MS, m/z 619.5 M^+1^[620] percent, calcd: C, 61.99; H, 2.93; N, 11.30%, percent, found: C, 61.65; H, 2.80; N, 11.02%. FT-IR (cm^−1^) 3358 (OH), 2190 (CN), 1726 (2 C = O), 1585(C = N), 1510 (N = N), 1486 (C-O); ^1^HNMR (d, ppm), 3.713 (s, 6 H, 2OCH_3_), 4.774 (s, H, CH chromone), 6.083–7.954 (m, 13H, 2C_6_H_4_, C_6_H_3_, C_6_H_2_), 10.03 (s, 1H, OH). ^13^C NMR (101 MHz, DMSO*d*6) δ (ppm):29.9, 55.8,77.9, 105.7,113.2, 114.8, 114.8, 117.3, 119.8, 122.3, 122.3, 122.3, 124.5,125.3, 125.8,129.2, 129.2, 129.4, 132.6,135, 135, 135.7, 140, 140, ,151.5, 156.7, 159, 160.8, 160.8, 170.8.

Dye**3:-6-((2-chloro-4-nitrophenyl) diazenyl)-4-(3**,**4-dimethoxyphenyl)-7-hydroxy-2-((E)-(4-hydroxy-3-methoxybenzylidene) amino)-4 H-chromene-3-carbonitrile**.

Dark brown powder, m.p. 120 °C, yield (82%), C_32_H_24_N_5_O_8_Cl MS, m/z 641.5 M^+^[641] percent, calcd: C, 59.87; H, 3.77; N, 10.91%, found: C, 59.57; H, 3.60; N, 10.71 per cent. FT-IR (cm^−1^)3278 (2OH), 2190 (CN), 1588 (C = N), 1507 (N = N), 1462 (C-O); ^1^HNMR (d, ppm), 3.69 (s, 9 H, 3OCH_3_), 4.53 (s, H, CH chromone), 6.37–7.04 (m, 12 H, C_6_H_4_, 2C_6_H_3_, C_6_H_2_), 7.057 (s, 1H, Sciff base C = N), 9.74 (s, 2 H, 2OH).^13^C NMR (101 MHz, DMSO*d*6) δ (ppm): 30.2, 56.1, 56.1, 56.1, 77.9, 105.7,112.0, 112.1,112.9 113.2, 117.0, 117.3, 119.8, 121.5, 122.3, 124.5, 125.3,125.8, 127.3, 129.4, 133.6, 149.3,150.3,150.4, 151.0, 151.5, 156.7, 158.0, 163.7, 170.8.

Dye **4:-6-((2-chloro-4-nitrophenyl)diazenyl)-4-(3**,**4-dimethoxyphenyl)-2-((1**,**3-dioxo-1 H-inden-2(3 H)-ylidene)amino)-7-hydroxy-4 H-chromene-3-carbonitrile**.

Brown powder, m.p. 110 °C, yield (77%), C_33_H_20_N_5_O_8_Cl MS, m/z 649.5 M^+^[649] percent, calcd: C, 60.98; H, 3.10; N, 10.77%, found: C, 60.77; H, 3.09; N, 10.70FT-IR (cm^−1^) 3358 (OH), 2190 (CN), 1726 (2 C = O), 1585(C = N), 1510 (N = N), 1486 (C-O); ^1^HNMR (d, ppm), 3.713 (s, 9 H, 3OCH_3_), 4.774 (s, H, CH chromone), 6.083–7.954 (m, 13H, 2C_6_H_4_, C_6_H_3_, C_6_H_2_), 10.03 (s, 1H, OH). ^13^C NMR (101 MHz, DMSO*d*6) δ (ppm): 30.2, 56.1, 56.1, 77.9, 105.7, 112.0, 112.9, 113.2, 117.3, 119.8, 121.5, 122.3, 122.3, 122.3, 124.5,125.3, 125.8, 129.4,133.6, 135, 135, 135.7, 140, 140, 150.3, 150.4,151.5, 156.7, 159, 160.8, 160.8, 170.8.

### Dyeing procedures, color measurements, and fastness testing of the dyed fabrics

The dyeing process is performed using two different techniques: ultrasonic and high temperature-high pressure (IR). The dyeing procedures of all dyes used were applied at a 1:50 liquor ratio with nylon and polyester fabrics.

#### High temperature-high pressure dyeing technique (HTHP)

A dispersion of all dyes 1–4 was produced by dissolving the appropriate amount of dye in drops of DMF (Liquor ratio 1:50) while studying the other dyeing factors such as dispersing agent, concentration, pH, time, and temperature for both fabrics.

After the coloring procedures, the dyed samples were taken out, thoroughly rinsed in warm water, and then reduced in a solution containing 2 g/L of sodium hydrosulphite and 2 g/L of sodium hydroxide (caustic soda) for 10 min at 60 °C with a 1:40 liquor ratio. After removing the dyed samples, they were washed with tap water and left to dry outside^22^.

#### Ultrasound dyeing

All dyes 1–4 were applied at 80 °C, and a 50:1 liquor ratio, with studying the other dyeing factors dispersing agent, shade, pH, and time for both fabrics, in addition to studying carrier as a factor for dyeing polyester fabric.

### Colour measurements

Using a D65/108 source and barium sulphate as the standard blank, The color strength (k/s) values, reflectance (%), and CLE L*a*b* value, which represent the color depth and the color strength of dyed samples that were evaluated in terms of their CLE L*a*b* coordinates and k/s values, were calculated on a CE 7000 A reflectance spectrophotometer (Gretag Macbeth, UK). The specular component was included with UV elimination and three average settings from repeated measurements^23,24^. The CIE 1976 Color Space technique was used to express the color values. The color values were determined through the following coordinates: L* for lightness, a* for greenness (negative value) and redness (positive value), b* for blueness (negative value) and yellowness (positive value), C* for chroma and h* for hue angle. We can measure the reflectance and colour strength from Eq. 1:

1 $$\text{K/S} = (1-\text{R}) 2/2\text{R}$$

Where K act as absorbance, S for scattering, and R for reflectance. Different samples’ colour strengths were assessed using the equation above.

### Fastness testing

The dyed samples were tested after washing off using 2 g/l non-ionic detergents at 80 °C for 30 min and were tested for their wash fastness, light fastness, rub fastness, and perspiration fastness according to ISO standard methods. Wash fastness (ISO 105-C02 (1989), crock fastness (ISO 105-X12 (1987), and perspiration fastness (ISO 105-E04 (1989)) were all evaluated visually using the ISO Gray Scale for both color change (AATCC Evaluation Procedure (EP) 1-similar to ISO 105-A02) and color staining (AATCC EP 2—same as ISO 105-A03). ISO 105-B02 was used to assess light fastness (carbon arc)^25–27^.

### Evaluation of antimicrobial activity

#### Materials

Two bacterial strains were E. coli ATCC 11,229 (gram-negative) and S. aureus ATCC 6538 (gram-positive). In addition, two fungal strains were Aspergillus nigra and Candida albicans. These bacterial strains were selected as test cells because they are the most frequent bacteria in wound infections. Fresh inoculants for antibacterial assessment were prepared in nutrient broth at 37 °C for 24 h.

#### Test method

For this investigation, the agar colony counting method was Applied^21^. The antimicrobial activities of the evaluated compounds were tested against S. aureus as gram-positive bacteria, E. coli as gram-negative bacteria, and two fungi, Aspergillus Niger and Candida albicans. A liquid culture was prepared by mixing 0.5 g peptone and 0.3 g beef extract in 100 ml water. 1 cm of fabric was cut and put into 10 ml of liquid culture, to which 10 µl of microbe culture was inoculated. All samples were incubated for 24 h at 37 °C. From each incubated sample, 100 µl of solution was taken, diluted, and distributed onto an agar plate. All plates were incubated for 24 h, and the colonies formed were counted. The percentage of bacteria reduction was determined as follows:

$$\text{Reduction in CFU (colony-forming units)}: \%= (\text{C}-\text{A})/\text{C} \times 100.$$

Where A is CFU/ml after contact (end test), and C is CFU/ml at zero contact time.

## Result and discussion

### Synthesis and spectroscopic characterization

Scheme 1 shows the structures of investigated compounds **a** and **b**, which were confirmed by elemental analysis (Experimental section), FT-IR, and ^1^HNMR. Spectra of **a** and **b** revealed the absorption bands localized within 1379–1370 cm-1 correspond to CO of chromene aromatic stretching. These stretching vibrations of C = C aromatic appeared at 1550 cm-1, a sharp CN absorption around 2199–2190 cm-1, NH2 stretching at about 3325 –3321 cm-1, and OH absorption band at 3343 − 3340 region. Compounds **a** and **b are** displayed in their ^1^HNMR singlet (3 H) at 3.803 ppm integrating for CH_3_ of compound **a** and singlet (6 H for 2CH) at 3.823 for compound **b**. singlet signals at 4.47 ppm are due to the CH of chromene protons. Multiple signals are due to aromatic rings. A singlet signal is due to NH_2_ at 8.22. A singlet signal at 9.8 is due to OH. Schemes 2 and 3 show that the Schiff base is formed by the structures of the investigated compounds **av**,** bv**,** an**, and **bn.** Mass spectra of the Schiff base compounds revealed molecular ions at m/z 428 [M+], 458 [M+], 436 [M+], and 467 [M + 1] for compounds **av**,** bv**,** an**, and **bn**,** respectively**. All structures were illustrated by elemental analysis (the experimental part). The IR spectra of all compounds showed C = N stretching modes of the Schiff base around 1559–1500. Compounds av and bv are characterized by the appearance of stretching mode OH of vanillin compounds and **an**,** and bn** are characterized by the appearance 2 C = O of ninhydrin at 1600. ^1^HNMR spectra of **av**,** bv**,** an**, and **bn** were observed at their respective fields. The singlet signal of NH_2_ disappears, and the presence of a singlet signal of Schiff base in compounds **av** and **bv**. Scheme 4 shows the structure of dyes **1**,** 2**,** 3**, and **4** confirmed by elemental analysis (exp. Part). IR spectra of all dyes showed the presence of N = N stretching mode of azo dyes at 1589 –1553 cm-1.

### High temperature-high pressure technique

#### Effect of dispersing agent

**Fig. 1:**
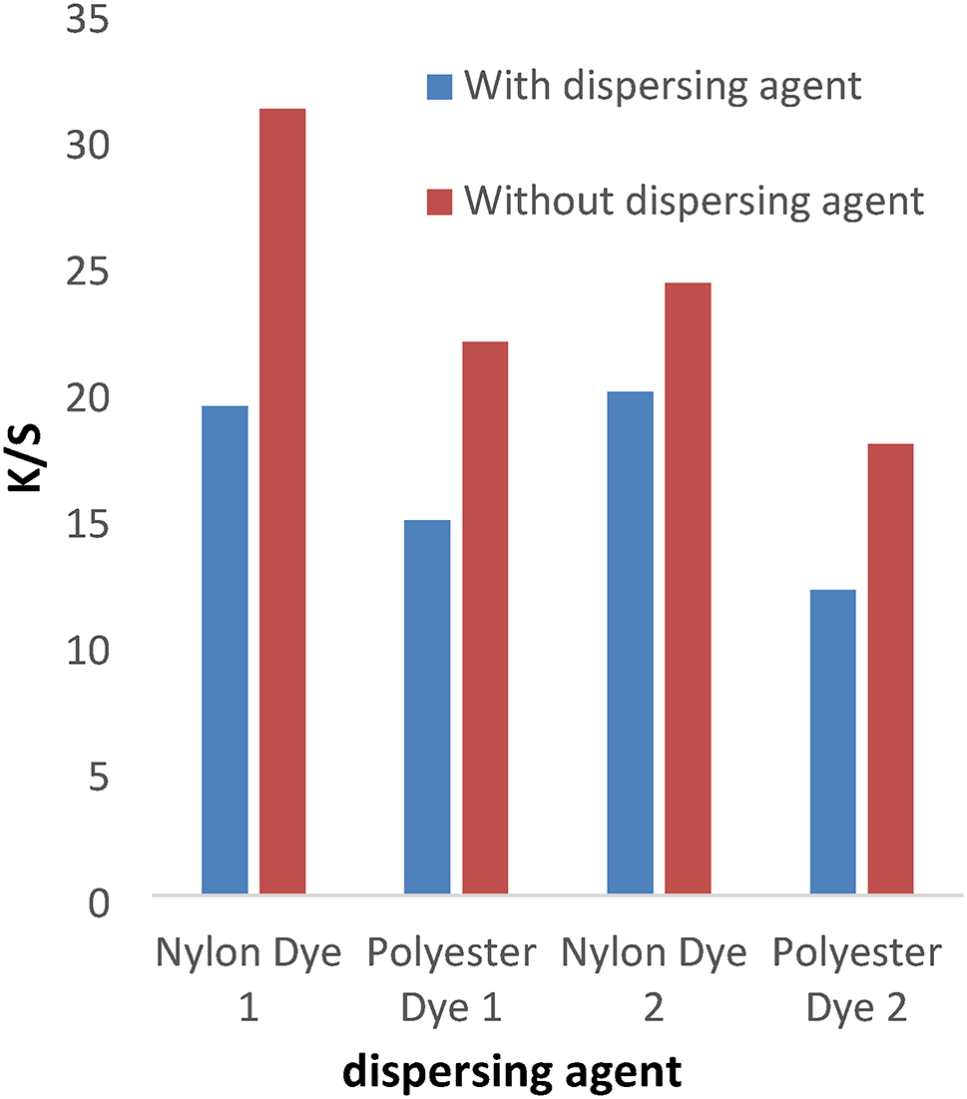
Effect of dispersing agent with K/S values in IR dyeing at PH = 4 for 1 h at 120 °C for dyes 1 and 2.

**Fig. 2:**
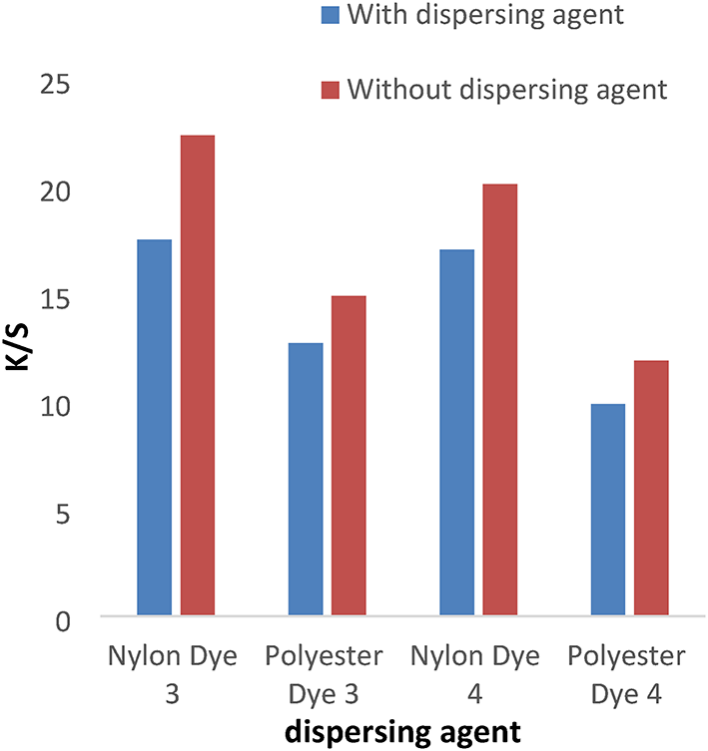
The effect of dispersing agent with K/S values in IR dyeing at PH = 4 for 1 h at a temperature of 120 °C for dyes 3 and 4.

The disperse dyes (1–4) were applied with or without the dispersing agent at PH = 4 for 1 h at a temperature of 120 °C for nylon and polyester fabrics. In Figs. 1 and 2, the data shows that the color intensity on nylon and polyester fabrics is higher without a dispersing agent than with a dispersing agent. Also, the data proved that the K/S for dyes 1 and 2 is higher than 3 and 4 due to the molecular weight of compounds (1 and 2) being lower than compounds (3) and 4. Disperse dyes have a tiny, planar, non-ionic structure with polar functional groups (CN) attached. The polar groups enhance the dye’s water solubility and dipolar bonding with the polymer, as well as changing the dye’s color. As well as the k/s values for polyester and nylon fabric colored with disperse dyes (1) and (3) are higher than those for dyes (2) and (4), respectively, due to the presence of a vanillin moiety in the Schiff base, which has more electron resonance than the ninhydrin moiety, which raises the color to the highest values. Also, the presence of the hydroxyl group in the vanillin moiety gives dyes more solubility than the ninhydrin moiety, which increases the diffusion of the dye particles into the fabrics.

#### Effect of concentration

A series of dyeing processes using disperse dyes (1–4) were carried out at various shades (1%, 2%, and 3%) at PH = 4 at 120 °C for 1 h without dispersing agents. The results of reflectance and K/S were estimated in Table 1. A higher shade percentage (%) also means a stronger dye absorption by the fabrics, which lowers the reflectance (%) value. In addition, a smaller shadow percentage (%) means that the substrate absorbs less dye, which raises the reflectance (%) value. Increasing the reflectance (%) value also affected the dyed fabric’s color strength (K/S). A relationship between shade (%), reflectance (%), and color strength (K/S) was discovered in this investigation. Reflectance (%) decrease with increase color strength (K/S). Concurrently, as shade (%) increase, color strength (K/S) increase and reflectance (%) decrease.

**Table 1:** Indicate the effect of concentration of disperse dyes **(1–4**) with K/S and reflectance values in IR dyeing at various shades (1%, 2%, and 3%) PH = 4 at 120 °C for 1 h without dispersing agents.

Dye no.	Shade (%)	Fabric	Reflectance (%)	K/S
DYE 1	1	N	3.05	15.40
		P	4.36	10.50
	2	N	2.93	17.17
		P	4.23	21.25
	3	N	2.19	31.11
		p	2.25	21.89
DYE 2	1	N	3.41	13.67
		P	10.79	8.28
	2	N	2.45	23.18
		P	5.41	17.86
	3	N	2.07	24.23
		p	11.90	25.31
DYE 3	1	N	4.41	10.37
		P	7.69	17.98
	2	N	3.72	16.47
		P	4.24	14.89
	3	N	2.86	22.86
		p	2.64	5.30
DYE 4	1	N	3.45	13.53
		P	4.66	15.38
	2	N	3.12	17.2
		P	4.05	11.89
	3	N	2.36	20.09
		p	3.27	10.26

#### Effect of pH

**Fig. 3:**
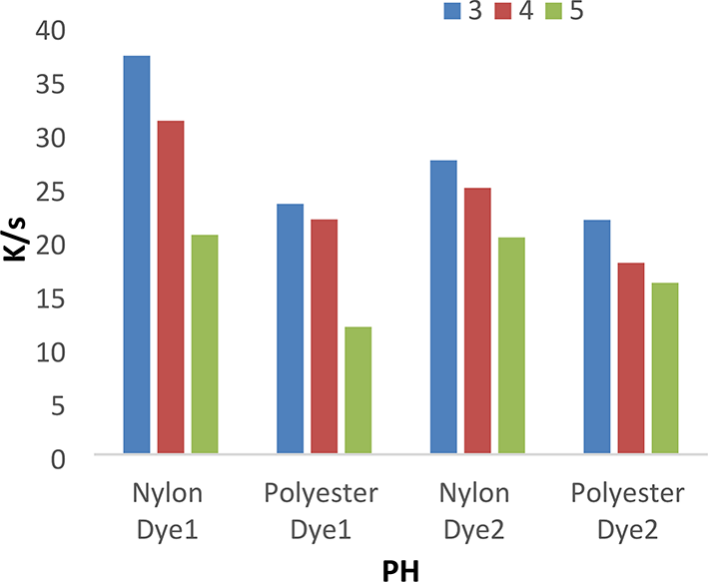
The effect of PH with K/S values in IR dyeing without dispersing agent for 1 h at 120 °C.

**Fig. 4:**
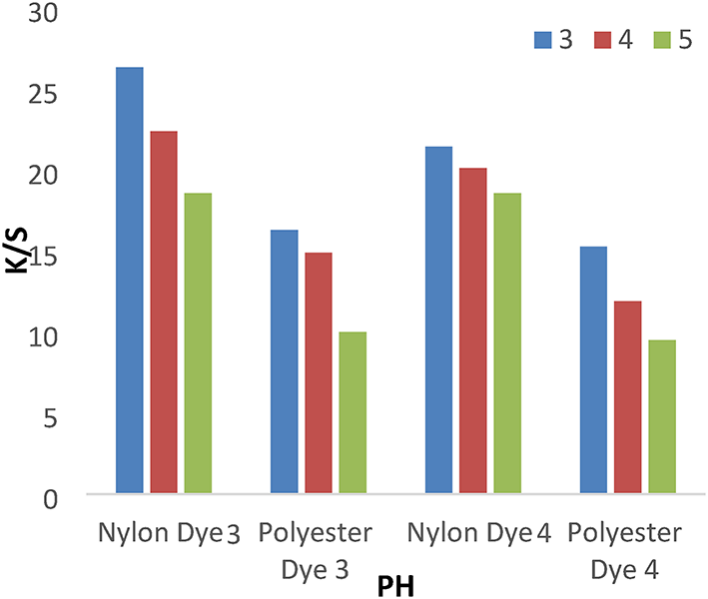
The effect of PH with K/S values in IR dyeing without dispersing agent for 1 h at 120 °C.

A series of dyeing processes using disperse dyes **(1–4**) were carried out at various dyebath pH (3–5) at 120 °C for 1 h without dispersing agents. The results shown in Figs. 3 and 4 indicated that pH 3 achieves higher K/S values than 4 and 5. We used a pH of 4 for all investigated values, which does not cause any degradation of fibers or dyes at high temperatures.

#### Effect of temperature

**Fig. 5:**
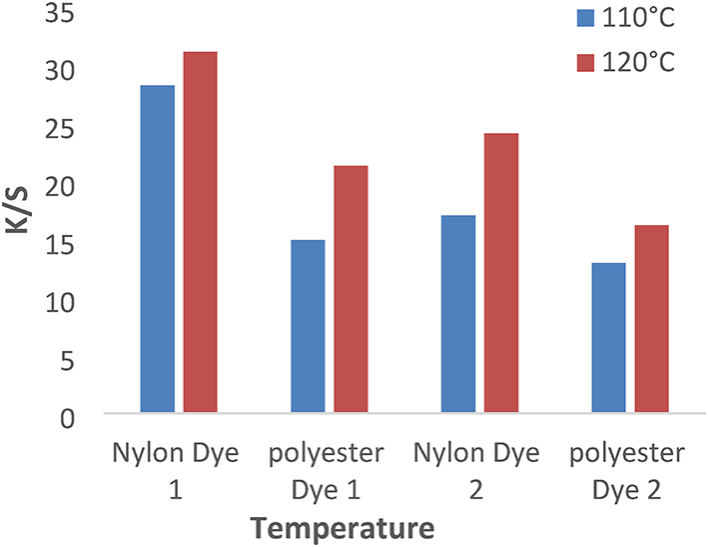
The effect of temperature with K/S values in IR dyeing without dispersing agent for 1 hour at PH 4 for dyes 1 and 2.

**Fig. 6:**
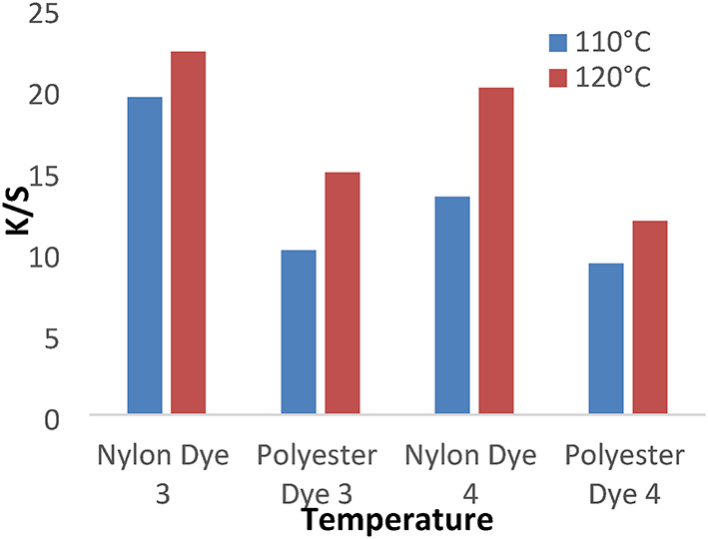
The effect of temperature with K/S values in IR dyeing without dispersing agent for 1 hour at PH 4 for dyes 3 and 4.

The study we extended to examine the dyeing behavior of disperse dyes **(1–4**) at different dyeing temperatures110 and 120 °C for 1 h at pH 4 without dispersing agents. Figures 5 and 6 showed that the temperature of 120 °C is higher K/S values for both polyester and nylon fabrics than other temperature values that are related to the physical and chemical properties of polyester and nylon fiber and the solubility of disperse dyes.

#### Effect of time

**Fig. 7:**
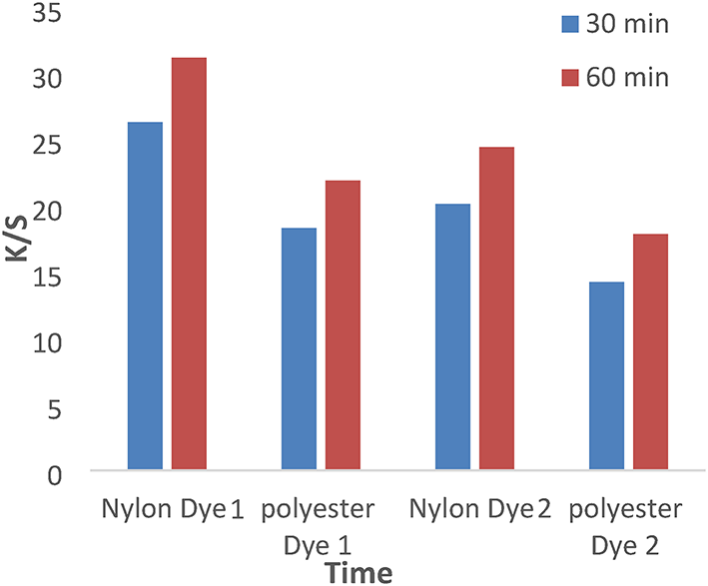
The effect of time with K/S values in IR dyeing without dispersing agent for 1 h at PH 4 for dyes 1 and 2.

**Fig. 8:**
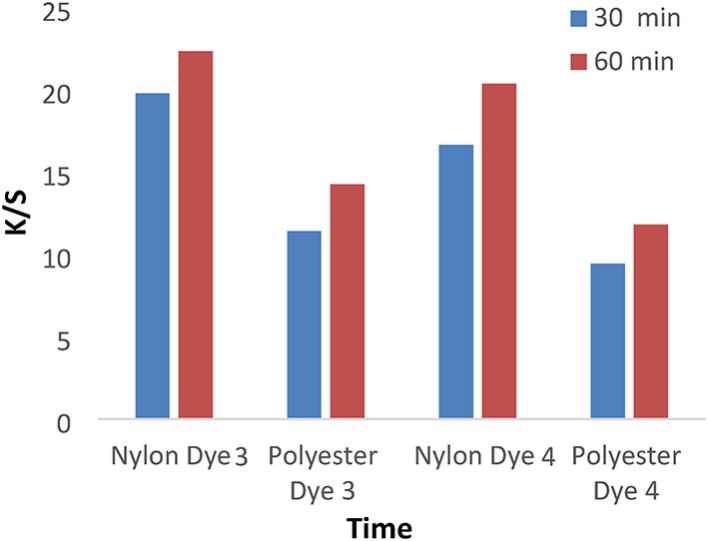
The effect of time with K/S values in IR dyeing without dispersing agent for 1 h at PH 4 for dyes 3 and 4.

Figures 7 and 8 show the dyeing behavior of disperse dyes (1–4) at different dyeing times (30 and 60 min) at temperatures of 120 °C and a pH of 4 without dispersing agents. However, the dyeing rate for all investigated dyes (1, 2, 3, and 4) is high at 30 min, with the highest k/s values achieved at 60 min due to the dyeing tendency to equilibrate after 60 min.

### Ultrasonic technique

#### Effect of dispersing agent

**Fig. 9:**
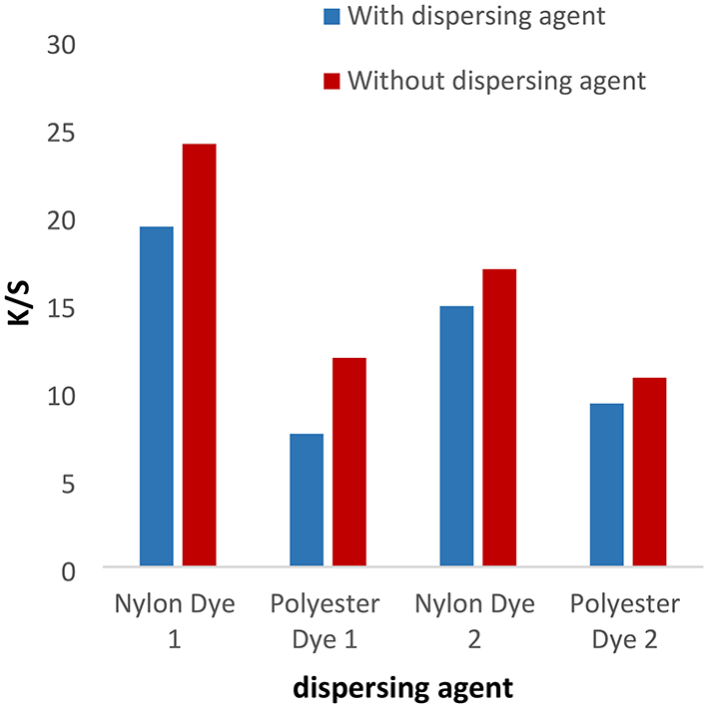
The effect of dispersing agents with K/S values in IR dyeing at PH = 4 for 1 h at 80 °C for dyes 1 and 2.

**Fig. 10:**
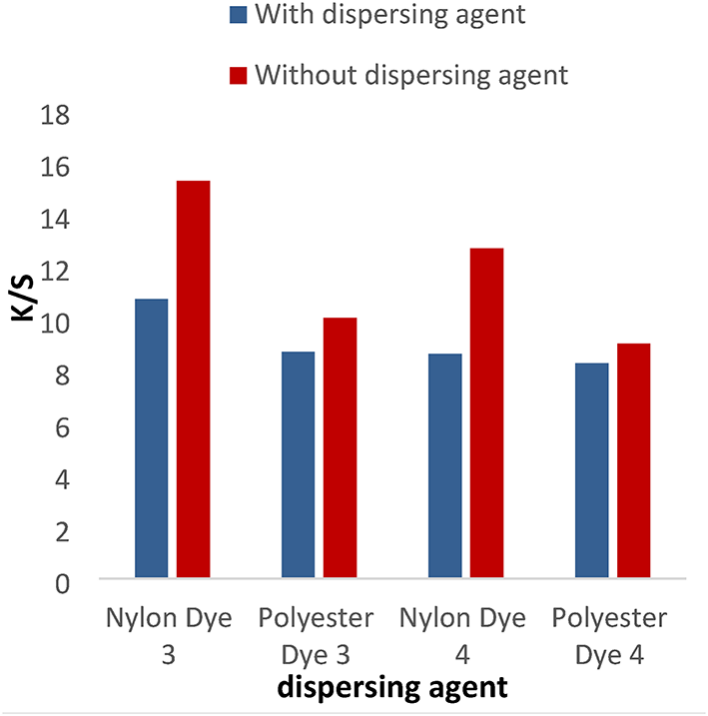
The effect of dispersing agents with K/S values in ultrasonic dyeing at PH = 4 for 1 h at 80 °C for dyes 3 and 4.

The disperse dyes (**1–4**) were applied with or without a dispersing agent at a pH of 4 for 1 h at a temperature of 80 °C for nylon and polyester fabrics. According to Figs. 9 and 10, the color intensity of dyed samples (1–4) (nylon and polyester fabrics) reached its highest values when dispersion agents were not used, compared to when they were. Furthermore, the data demonstrated that the K/S for dyes 1 and 3 is higher than that of 2 and 4, respectively, as previously discussed.

#### Effect of concentration

A series of dyeing using disperse dyes (1–4) were carried out at various shades (1%,2%, and 3%) at PH = 4 at 80 °C, for 1 h without dispersing agents. The results of reflectance and K/S were estimated as shown in Table 2. In this experiment, the data revealed a link between shade (%), reflectance (%), and color strength (K/S) was found. As shade (%) rises, color strength (K/S) increase. On the other hand, the color strength (K/S) increase then the reflectance (%) decrease 0.

**Table 2:** Indicates the effect of concentration of disperse dyes **(1–4**) with K/S and reflectance values in Ultrasonic dyeing at various shades (1%, 2%, and 3%) PH = 4 at 80 °C for 1 h without dispersing agent.

Dye no.	Shade (%)	Fabric	Reflectance (%)	K/S
DYE 1	1	N	3.04	15.46
		P	9.44	7.12
	2	N	2.9	17.80
		P	6.16	9.44
	3	N	2.66	24.08
		p	4.90	11.9
DYE 2	1	N	4.43	10.30
		P	6.65	9.93
	2	N	3.89	16.95
		P	6.02	10.11
	3	N	2.32	20.58
		p	4.15	11.07
DYE 3	1	N	3.86	11.99
		P	6.05	7.02
	2	N	3.18	14.72
		P	5.56	7.86
	3	N	2.69	17.62
		p	4.99	10
DYE 4	1	N	5.69	7.82
		P	6.98	6.19
	2	N	4.35	10.68
		P	5.87	8.01
	3	N	3.96	12.68
		p	5.01	9.02

#### Effect of pH

**Fig. 11:**
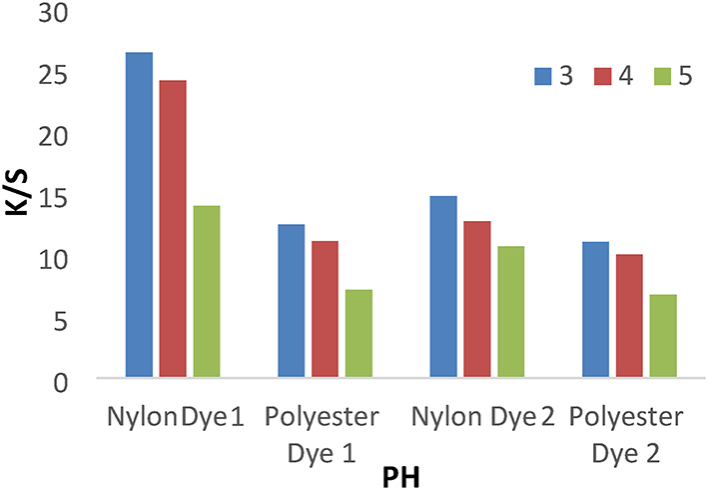
The effect of pH with K/S values in ultrasonic dyeing without dispersing agent for 1 h at 80 °C.

**Fig. 12:**
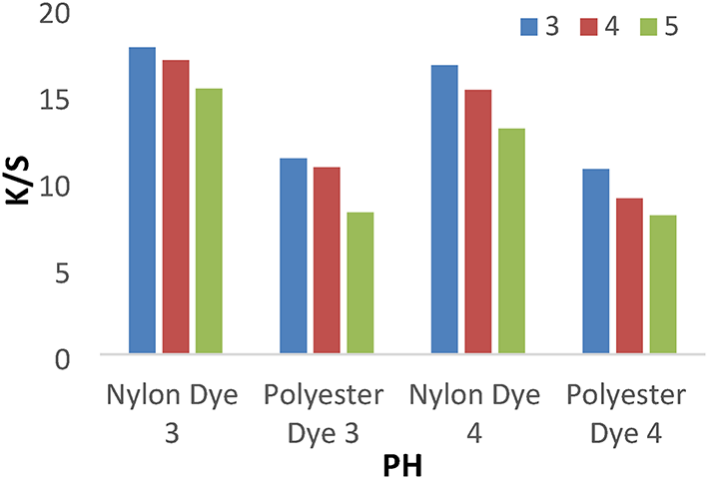
Effect of pH with K/S values in ultrasonic dyeing without dispersing agent for 1 h at 80 °C.

A series of dyeing using disperse dyes **(1–4**) were carried out at various dyebath pH (3–5) at 80 °C for 1 h without dispersing agents. The results shown in Figs. 11 and 12 indicated that pH 4 achieves higher K/S values than 3 and 5. As it’s a suitable pH for both fabric and dye, it doesn’t cause any hydrolysis for the fabric or decomposition of azo disperse dyes.

#### Effect of time

**Fig. 13:**
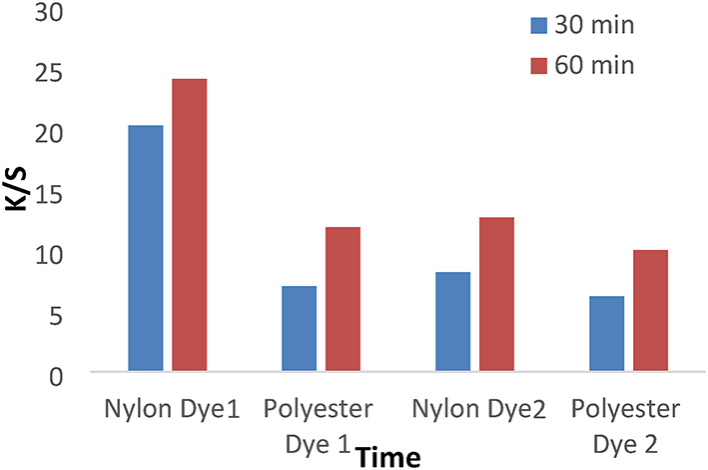
The effect of time on K/S values in ultrasonic dyeing without a dispersing agent for 1 h at PH 4 for dyes 1 and 2.

**Fig. 14:**
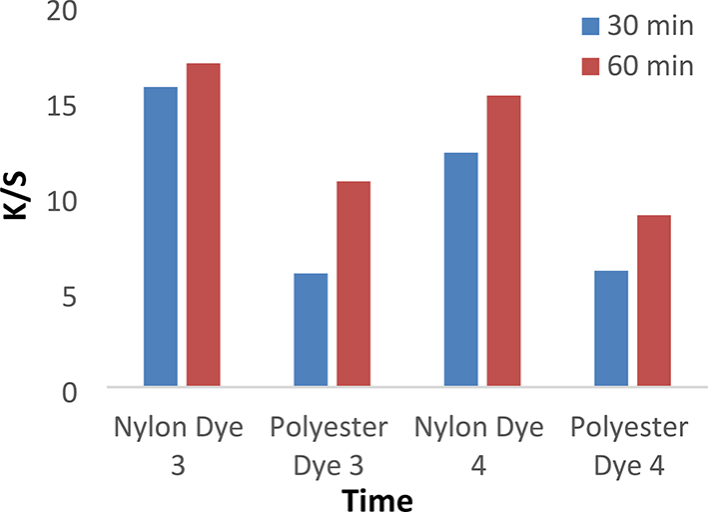
Effect of time with K/S values in ultrasonic dyeing without dispersing agent for 1 h at PH 4 for dyes 1 and 2.

Figures 13 and 14 show the dyeing behavior of disperse dyes **(1–4**) at different dyeing times of 30 and 60 min at temperatures of 80 °C and pH = 4 without dispersing agents. However, the dyeing rate for all investigated dyes (1, 2, 3, and 4) is high at 30 min, with the highest k/s values achieved at 60 min due to the dyeing tendency to equilibrate after 60 min.

#### Effect of carrier

**Fig. 15:**
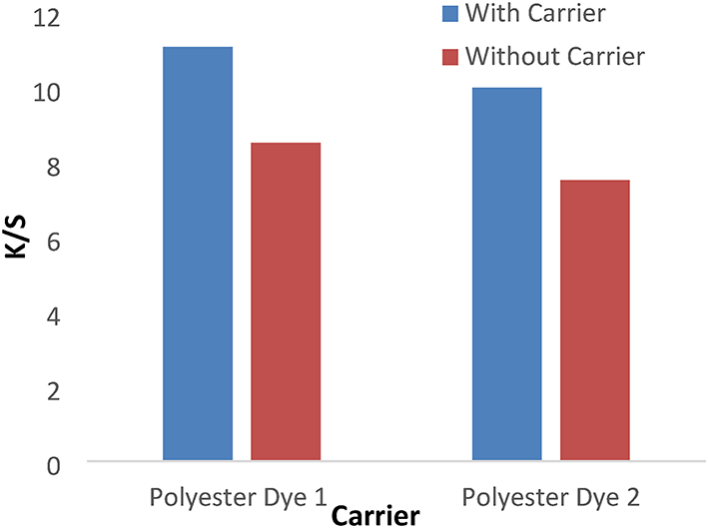
The effect of the carrier with K/S values in ultrasonic dyeing without dispersing agent for 1 h at PH 4 for dyes 1 and 2 for polyester.

**Fig. 16:**
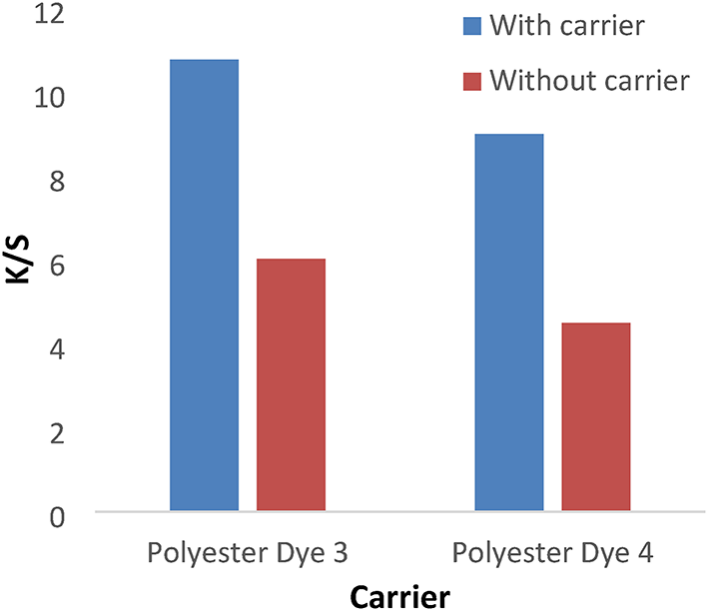
The effect of the carrier with K/S values in ultrasonic dyeing without dispersing agent for 1 h at PH 4 for dyes 3 and 4 for polyester.

Figures 15 and 16 show the ultrasonic dyeing of polyester with a carrier has a higher K/S than without a carrier. Using a carrier enhances the dye’s adsorption and diffusion into the fibers during the dyeing process, as polyester materials require temperatures above 125 °C to achieve adequate color penetration into the fabric’s structure.

### Colorimetric and fastness properties

Data show that dyes 3 and 4 are higher in light fastness than dyes 1 and 2 due to the presence of two groups of OCH_3_, which increase the resonance of electrons and thus increase the light fastness. Also, IR dyeing techniques have higher light fastness than ultrasonic techniques. It should be highlighted that no color shift was seen, and that very good wash fastness ratings(4–5) and very good rubbing fastness ratings(4–5) were obtained in both wet and dry conditions. Perspiration fastness properties (in both acidic and alkaline media) of dyed polyester and nylon samples in terms of ratings for staining of adjacent fabrics and changing in color were very good (Table 3).

**Table 3:** Indicates the fastness properties of dyed polyester and nylon at 120 ˚C for dyes **1**,** 2**, 3, and 4 without dispersing agent at the I.R. technique and without dispersing agent at 80 ˚C using ultrasonic techniques.

		Properties Fabric	Light	Rubbing		Washing					Perspiration									
											Alkaline					Acidic				
				Wet	Dry	Alt	SC	SW	SP	SN	Alt	SC	SW	SN	SP	Alt	SC	SW	SN	SP
**DYE 1**	IR	Nylon	4	3	4	4–5	4–5	4	4–5	3–4	4–5	4	4	3	4–5	4–5	4–5	4	3	4–5
		Polyester	5	4	4–5	4–5	4–5	4–5	4–5	4–5	4–5	4–5	4–5	4–5	4–5	4–5	4–5	4–5	4–5	4–5
	Ultrasonic	Nylon	4	3–4	3–4	4–5	4–5	3–4	3–4	3	4–5	4	4	3	4–5	4–5	4–5	4–5	4–5	4–5
		Polyester	4	4	4	4	4	3–4	3–4	3	4–5	3	3	3	3	4–5	4	3	3	4–5
**DYE 2**	IR	Nylon	5–6	5	5	4–5	4–5	4–5	4–5	4–5	4–5	4–5	4–5	4–5	4–5	4–5	4–5	4–5	4–5	4–5
		Polyester	6–7	5	5	4–5	4–5	4–5	4–5	4–5	4–5	4–5	4–5	4–5	4–5	4–5	4–5	4–5	4–5	4–5
	Ultrasonic	Nylon	4–5	3	4	4–5	4–5	4	4	4	4–5	4–5	4–5	4–5	4–5	4–5	4–5	4–5	4–5	4–5
		Polyester	5	3–4	3–4	4–5	4–5	4–5	4–5	4–5	4–5	4	4	4	3	4–5	4–5	4	4	3
**DYE 3**	IR	Nylon	4	4–5	4–5	4–5	4–5	4	4–5	3–4	4–5	4	4	4	4–5	4–5	4	4	4	4–5
		Polyester	4	4–5	4–5	4–5	4–5	4–5	4–5	4–5	4–5	4–5	4–5	4–5	4–5	4–5	4–5	4–5	4–5	4–5
	Ultrasonic	Nylon	4	4–5	4–5	3–4	4–5	3–4	4–5	3	4–5	3	4	3	4–5	4–5	4–5	4	3–4	4–5
		Polyester	4	4–5	4–5	4–5	4–5	4–5	4–5	4–5	4–5	4–5	4–5	4–5	4–5	4–5	4–5	4–5	4–5	4–5
**DYE 4**	IR	Nylon	5–6	4	4	4–5	4–5	4–5	4–5	4–5	4–5	4–5	4–5	4–5	4–5	4–5	4–5	4–5	4–5	4–5
		Polyester	6–7	4–5	4–5	4–5	4–5	4–5	4–5	4–5	4–5	4–5	4–5	4–5	4–5	4–5	4–5	4–5	4–5	4–5
	Ultrasonic	Nylon	4–5	3–4	4	4–5	4–5	4–5	4–5	3–4	4–5	4–5	4–5	4–5	4–5	4–5	3	4–5	4	4–5
		Polyester	5	4	4	4–5	4–5	4–5	4–5	4–5	4–5	4–5	4–5	4–5	4–5	4–5	4–5	4–5	4–5	4–5

Also, the color parameters were evaluated using the Cielab system and the modified CIE L* C * h^o^ (D65/10^o^) system (Table 4). The following color parameters for the dyed samples were obtained by the digital Cielab system: L* – lightness, a* – redness if positive coordinate, or greenness if negative coordinate, b* – yellowness if positive coordinate, or blueness if negative coordinate, C* – chromaticity, h – hue of the color, X – coordinate x, Y – coordinate y, Z – coordinate z (McDonald, 1997).

**Table 4:** Shows the colorimetric data of dyed polyester and nylon with dyes **1**,** 2**,** 3**, and **4** at 80 ˚C without dispersing agents using ultrasonic techniques and at 120 ˚C without dispersing agents using the I.R. technique.

Dye	ƛmax	Technique Of dyeing	Properties Fabric	L*	a*	b*	dE*	dH*
**DYE 1**	**395**	IR	Nylon	31.11	43.69	27.36	39.71	63.77
			Polyester	21.89	31.07	18.07	18.68	56.77
		Ultrasonic	Nylon	24.08	40.49	30.51	37.34	65.21
			Polyester	11.9	52.54	14.29	27.07	44.80
**DYE 2**	**420**	IR	Nylon	24.23	37.57	15.59	36.13	59.60
			Polyester	17.86	47.61	32.81	53.91	74.00
		Ultrasonic	Nylon	12.68	49.89	7.17	37.11	53.26
			Polyester	10.0	52.17	19.75	36.26	52.65
**DYE 3**	**390**	IR	Nylon	22.36	39.55	15.43	24.82	51.43
			Polyester	14.89	56.37	9.07	33.81	47.42
		Ultrasonic	Nylon	16.95	42.34	19.76	34.64	58.96
			Polyester	10.77	54.25	13.43	33.45	49.42
**DYE 4**	**41 0**	IR	Nylon	20.09	41.96	16.03	40.61	61.86
			Polyester	11.89	51.55	28.73	52.76	71.13
		Ultrasonic	Nylon	15.26	54.65	4.60	38.98	51.82
			Polyester	9.0	51.59	16.43	37.33	54.72

### Antimicrobial activity

The antibacterial efficacy of the prepared derivatives was evaluated through a wide spectrum using the colony counting method applied^21^, as pronounced in the Experimental section. Some positive controls were employed to evaluate the activity of the dyed fabrics using synthesized derivatives against both different bacterial strains, such as S. aureus as a gram-positive bacteria, E. coli as a gram-negative bacteria, and two fungi, Aspergillus niger, and Candida albicans.

Initially, all of the synthesized dyes (1, 2, 3, and 4) exhibited appropriate antimicrobial efficacy towards different types of microbes, with percentage reductions in colony-forming units ranging from around 57% to nearly 100% due to the presence of hydroxyl and carbonyl groups. Meanwhile, the carbonyl groups in Dye 2 demonstrate higher reduction results toward S. aureus colonies compared to the reduction results of coli colonies. Likewise, Dye 4 contains two carbonyl groups, exhibiting excellent antibacterial performance comparable to that of Dye 2 across all dyeing conditions and fabric types. In contrast, dyes 1 and 3 exhibited moderate to good antimicrobial activity, slightly lower than dyes 2 and 4, according to the chemical structure of dyes 2 and 4, while they have carbonyl groups bearing a positive charge on nitrogen atoms and thus, in turn, have an electrostatic interaction with the charge of microorganisms in the cell membrane, which is responsible for inhibiting the growth of bacteria and fungi.

The dyeing techniques that are employed on the fabrics also affect the antimicrobial activity results (Table 5). For example, the IR dyeing technique that is applied to all synthesized dyes (1, 2, 3, and 4) generally results in slightly better antimicrobial activity compared to the ultrasonic dyeing technique, especially for nylon fabric, as increasing the diffusion of the dye molecular inside the fabric increases the biological activity and effectiveness of dyed samples.

**Table 5:** Antimicrobial activity of dyed polyester and nylon fabrics with dyes **1**,** 2**,** 3**, and **4** at 80 ˚C without dispersing agents using ultrasonic techniques and at 120 ˚C without dispersing agents using the I.R. technique.

Sample No	S. aureus		E. coli		Aspergillus Niger		Candida albicans	
	Colonies No.	Reduction	Colonies No.	Reduction	Colonies No.	Reduction	Colonies No.	Reduction
	CFU x10^5^	%	CFU x10^7^	%	CFU x10^5^	%	CFU x10^7^	%
Blank	5.1	0	17.7	0	21.9	0	18.2	0
Dye 1P. ult	0.98	86.86	3.01	89.32	1.11	80.52	1.18	82.64
Dye 1 P. IR	0.67	86.86	1.89	89.32	3.1	85.84	1.29	92.91
Dye 1 N. IR	0.087	98.47	1.02	94.23	0.09	98.30	0.06	98.78
Dye 1 N ult	0.078	98.72	0.98	94.46	0.9	83.02	0.34	93.06
Dye 2 P. IR	0.66	89.80	1.65	90.67	0.87	83.58	0.82	83.27
Dye 2 P. ult	0.12	98.29	1.32	94.23	1.23	76.79	0.32	93.47
Dye 2 N. ult	0.021	99.70	0.77	95.64	0.09	98.30	0.06	98.78
Dye 2 N. IR	0.015	99.93	0.77	95.64	0.32	94.38	0.19	97.2
Dye 3 p. IR	1.67	79.80	7.57	57.23	10.2	53.42	7.57	58.41
Dye 3 P. ult	1.03	86.86	4.21	76.21	5.6	74.43	4.21	76.87
Dye 3 N. ult	0.67	87.05	1.89	89.32	8.1	63.01	2.11	88.41
Dye 3 N. IR	0.52	98.29	1.45	91.80	0.32	93.96	0.21	95.71
Dye 4 P. IR	0.065	98.92	0.77	95.64	0.3	94.34	0.09	98.16
Dye 4. P ult	0.065	98.9	0.77	95.64	0.09	98.30	0.06	98.78
Dye 4 N. ult	0.055	98.94	0.77	95.64	0.32	94.38	0.19	97.2
Dye 4 N. IR	0.054	99.58	0.77	95.64	0.32	93.96	0.21	95.71

Interestingly, the antimicrobial efficacy appeared to be slightly higher against the Gram-positive S. aureus compared to the Gram-negative E. coli and fungi (Aspergillus niger and Candida albicans) for all dyed fabrics with different dyeing techniques related to differences in cell wall structure and composition, as well as the interactions between dyes and microbial cells.

## Conclusion

The goal is to achieve simultaneous dyeing and antimicrobial finishing using new Schiff base azo disperse dyes based on chromene moiety. The k/s values of all dyed samples show good k/s values at temperatures (120 °C) for the high temperature-high pressure dyeing technique and (80 °C) for the ultrasonic dyeing technique in the continuous dyeing process for 1 h. Many different factors, such as dyeing technique, auxiliaries (like dispersing agents and carriers), shade, time, PH, and temperature, affect the physical and chemical properties of synthetic fabrics, especially polyester fabric, and the diffusion of dye molecules inside the fabric. The k/s values for polyester and nylon fabric colored with disperse dye (1) and (3) are higher than dye (2) and (4), respectively. Additionally, research on the effects of shadow on nylon and polyester fabrics revealed that color strength (K/S) and reflectance (%) decrease as shade (%) increases. Concurrently, as shade (%) decreases, reflectance (%) and color strength (K/S) rise. All dyed samples showed good fastness properties toward washing, light, perspiration, and rubbing, with a rating of (4–5) for staining of adjacent fabrics and changing color. Higher antibacterial properties against all tested bacteria (Pseudomonas aeruginosa, a gram-negative bacterium, and Staphylococcus aureus, a gram-positive bacterium) and fungi aspergillus Niger and Candida albicans are promoted by the presence of Schiff-based and chromene moiety in the examined dyes on nylon and polyester fabrics.

## Data Availability

Data is provided within the manuscript.
